# Smoking and Epstein–Barr virus infection in multiple sclerosis development

**DOI:** 10.1038/s41598-020-67883-w

**Published:** 2020-07-03

**Authors:** Anna Karin Hedström, Jesse Huang, Nicole Brenner, Julia Butt, Jan Hillert, Tim Waterboer, Ingrid Kockum, Tomas Olsson, Lars Alfredsson

**Affiliations:** 1 Department of Clinical Neuroscience, Karolinska Institutet, Stockholm, Sweden; 2 Institute of Environmental Medicine, Karolinska Institutet, Stockholm, Sweden; 3 Center for Molecular Medicine, Karolinska Institutet at Karolinska University Hospital, Stockholm, Sweden; 4 German Cancer Research Center (DKFZ), Heidelberg, Germany

**Keywords:** Epidemiology, Multiple sclerosis

## Abstract

It is unclear whether smoking interacts with different aspects of Epstein–Barr virus (EBV) infection with regard to multiple sclerosis (MS) risk. We aimed to investigate whether smoking acts synergistically with elevated EBNA-1 antibody levels or infectious mononucleosis (IM) history regarding MS risk. Two Swedish population-based case–control studies were used (6,340 cases and 6,219 matched controls). Subjects with different smoking, EBNA-1 and IM status were compared regarding MS risk, by calculating odds ratios (OR) with 95% confidence intervals (CI) employing logistic regression. Potential interaction on the additive scale was evaluated by calculating the attributable proportion due to interaction (AP). Current and past smokers had higher EBNA-1 antibody levels than never smokers (p < 0.0001). There was an additive interaction between current smoking and high EBNA-1 antibody levels (AP 0.3, 95% CI 0.2–0.4), but not between past smoking and high EBNA-1 antibody levels (AP 0.01, 95% CI − 0.1 to 0.1), with regard to MS risk. An interaction also occurred between current smoking and IM history (AP 0.2, 95% CI 0.004–0.4), but not between past smoking and IM history (AP − 0.06, 95% CI − 0.4 to 0.3). Current smoking increases EBNA-1 antibody levels and acts synergistically with both aspects of EBV infection to increase MS risk, indicating that there is at least one pathway to disease in which both risk factors are involved.

## Introduction

Multiple sclerosis (MS) is an inflammatory disease of the central nervous system with underlying genetic and environmental factors. Smoking, high levels of EBNA-1 Immunoglobulin G (IgG), and infectious mononucleosis (IM) history have consistently been associated with increased MS risk^1–3^.

Previous studies on the interplay between smoking and aspects of EBV infection have used different study designs and statistical methods, and sample sizes have often been limited which may have contributed to conflicting results (Table 1). The first study observed a positive interaction on the multiplicative scale between smoking and elevated EBNA-1 IgG levels with regard to MS risk^4^. Smoking was only observed to increase MS risk among subjects with high EBNA-1 IgG levels. These findings were not replicated in other studies^5–7^. A recent study showed a trend towards a negative interaction among young adults and a trend towards a positive interaction among older subjects, as defined by age at EBV assessment, suggesting age may influence the interaction between smoking and EBNA-1 IgG levels^8^.

**Table 1:** Studies on the potential interaction between smoking and measures of EBV.

Reference	No. of cases/controls	Exposure definitions	Interaction	Study design, comments
4	442/865	EBNA1 antibody levels Ever/never smoking at diagnosis	Positive multiplicative interaction (p value 0.0001) Additive interaction not tested	Pooled analyses from two case–control studies (Sweden and Tasmania) and one prospective study (United States)
5	662/848	EBNA1 antibody levels Ever/never smoking before disease onset	No multiplicative interaction Additive interaction, AP − 0.04 (− 0.4 to 0.3)	Swedish population-based case–control study
6	1,237/488	EBNA1 antibody levels Ever/never smoking before disease onset	No multiplicative interaction Additive interaction not tested	Case–control study (Accelerated Cure Project for Multiple Sclerosis)
7	206/217	EBNA1 antibody levels Ever/never smoking before disease onset	No additive or multiplicative interactions	Australian incident case–control study
8	192/384	EBNA1 antibody levels Cotinine levels < 10 or > 10 ng/ml at	No multiplicative interaction Additive interaction, AP 0.2 (− 0.2 to 0.6)	Swedish nested case–control study
6	1,237/488	IM history Ever/never smoking before disease onset	No multiplicative interaction Additive interaction not tested	Case–control study (Accelerated Cure Project for Multiple Sclerosis)
7	282/558	IM history Ever/never smoking before disease onset	No additive or multiplicative interactions	Australian incident case–control study
9	1904/3,694	IM history Ever/never smoking before disease onset	Negative multiplicative interaction (p = 0.001). Additive interaction [RERI − 0.98 (− 2.05 to 0.15)]	Case–control study (Italy, Norway, Sweden). Response rate among controls in each country was 36%, 37% versus 21%. Pooled analysis

Results from previous studies on a possible interaction between smoking and IM history have also been conflicting. Two studies found no interaction between smoking and IM history^6,7^, whereas a recent study reported a negative interaction on the multiplicative scale regarding MS risk^9^. Using two Swedish population-based case–control studies comprising 6,340 cases and 6,219 controls, we aimed to investigate the interplay between smoking and different aspects of EBV infection [elevated EBNA-1 IgG levels and infectious mononucleosis (IM) history] with regard to MS risk.

## Methods

The present report is based on two Swedish population-based case–control studies; Epidemiological Investigation of Multiple Sclerosis (EIMS) and Genes and Environment in Multiple Sclerosis (GEMS).The study base comprised the general population aged 16–70 years.

EIMS recruited incident cases of MS from neurology clinics throughout the country between April 2005 and June 2015. For each case included in the study, two controls were randomly selected from the national population register, frequency matched for the case's age in 5-year age strata, sex and residential area.

GEMS identified prevalent cases from the Swedish National MS-registry. Controls, matched for age, sex, and residential area at the time of disease onset, were recruited in the same manner as in EIMS. The study participants were recruited between November 2009 and November 2011. All cases in both studies fulfilled the McDonald criteria^10^. The studies were approved by the Regional Ethical Review Board at Karolinska Institutet and have therefore been performed in accordance with the ethical standards laid down in the 1964 Declaration of Helsinki and its later amendments. All participants provided informed consent.

### Data collection and exposure information

All participants in both studies answered a detailed questionnaire regarding environmental exposures and lifestyle factors. Questionnaires were completed by 2,880 cases and 6,122 controls in EIMS, and by 6,156 cases and 5,408 controls in GEMS. The response rate was 93% for cases and 73% for controls in EIMS, and 82% for cases and 66% for controls in GEMS. All participants were asked to provide a blood sample and those who did not were excluded. Blood samples were available for 2,021 cases and 2,449 controls in EIMS and for 4,319 cases and 3,770 controls in GEMS. The present study is thus based on 6,340 cases and 6,219 matched controls.

### Genotyping and measurement of EBNA-1 IgG levels

HLA-DRB1 and HLA-A alleles were determined at four-digit resolution. Genotyping was performed on the MS replication chip^11^ which is based on an Illumina exome chip to which approximately 90,000 custom markers were added with extra high density in the HLA region and HLA was then imputed with HLA*IMP:02^12^.

Multiplex serology was used for detection of IgG antibodies against the EBNA-1 peptide segment (aa 385–420)^13,14^, which has been identified as the primary EBNA-1 fragment associated with MS risk^15^. Dual-laser flow-based detection was used to quantify the antibodies as units of median flourescence intensity.

### Definitions of exposures

Participants were asked to provide information about current and previous smoking, including duration of smoking and average number of cigarettes smoked per day. The year of disease onset in the cases was defined as the index year and the corresponding controls were given the same index year. Smoking habits were only considered before the index year. Those who had smoked during the index year were defined as current smokers, those who had stopped smoking prior to the index year were defined as past smokers, and those who had never smoked before or during the index year were defined as never smokers.

EBNA-1 IgG levels were dichotomized based on the median seroreactivity among controls (5,620 MFI) defining groups with high and low EBNA-1 IgG levels. Based on self-reported information, IM history was dichotomized into yes or no. Subjects who could not provide information regarding a history of IM were excluded in the analysis on smoking and IM history (788 cases and 1,024 controls).

### Statistical analysis

Subjects categorized based on smoking status, EBNA-1 IgG levels and IM history were compared with regard to MS risk, by calculating odds ratios (OR) with 95% confidence intervals (CI) using logistic regression models.

Sufficient-cause interaction is present when two risk factors are involved in the same pathway towards disease. Rothman has shown that independent risk factors adhere on the additive model and that interaction results in departure from additivity of disease rates^16^. Since the logistic regression model is inherently multiplicative, presence of an interaction term implies departure from multiplicativity, which has no direct relevance for the issue of whether or not sufficient-cause interaction is present^17^. Interaction should thus preferably be assessed on the additive scale^18^.

Interaction on the additive scale between smoking and aspects of EBV infection (EBNA-1 status and IM respectively) was assessed by calculating the attributable proportion due to interaction (AP). In order to estimate the influence of age at EBV assessment on the potential interaction between smoking and EBNA-1 status, we performed the analyses stratified by age at EBV assessment, using the same cut-off as in a previous study^7^ (< 50 years or ≥ 50 years). Since interactions have been reported between the main genetic risk factor for MS, the DRB1*15:01 allele^19^, and both smoking^20^, high EBNA1 IgG^21^ and past IM^21^, we also stratified the analyses by DRB1*15:01 status.

All analyses were adjusted for age, sex, residential area, study, ancestry, adolescent body mass index (BMI), DRB1*03:01, DRB1*13:03, DRB1*08:01, A*02:01, B*44:02, B38:01, B44:02, DQA1*01:01, DQB1*03:01, and DQBI*03:02. Homozygote correction was made for DRB1*15:01, DRB1*03:01, and A*02:01. When appropriate we also adjusted for EBNA-1 status and past IM. Ancestry was dichotomized into Swedish or non-Swedish. Adolescent BMI was calculated by dividing self-reported weight in kilograms at age 20 years by self-reported height in meters squared and categorized into underweight (< 18.5 kg/m^2^), normal weight (18.5–25 kg/m^2^), overweight (25–30 kg/m^2^) and obese (> 30 kg/m^2^).

We additionally adjusted the analyses for passive smoking (yes or no), sun exposure habits (high or low), education (no post-secondary education, post-secondary education without university degree, or university degree), and socioeconomic index (workers in goods production, workers in service production, employees at lower/intermediate levels, employees at higher levels, and others), but these variables only had minor influence on the results and were not kept in the final analyses. These definitions have been described in previous publications based on the same studies^22–24^.

The proportion of missing data regarding IM history was 12% among cases and 16% among controls. We therefore conducted supplementary analyses after imputing missing data using the multiple imputation chained equation procedure^25^. We also conducted a sensitivity analysis in which smoking habits were considered 2 years prior to the index year. All analyses were conducted using Statistical Analysis System (SAS) version 9.4.

## Results

Our analyses regarding the interplay between smoking and different aspects of EBV infection with regard to MS risk included 6,340 MS cases and 6,219 controls. Characteristics of cases and controls, overall and by smoking status, are presented in online resource 1. Ever smoking was associated with increased MS risk regardless of EBNA-1 status (OR 1.4, 95% CI 1.3–1.6 among subjects with high EBNA-1 IgG and OR 1.4, 95% CI 1.2–1.7 among those with low EBNA-1 IgG). However, ever smokers had higher EBNA-1 IgG levels than never smokers, both among cases and controls (p values for significance between groups were < 0.0001).

There was an interaction on the additive scale between current smoking and high EBNA-1 IgG levels (AP 0.3, 95% CI 0.2–0.4), but not between past smoking and high EBNA-1 IgG levels (AP 0.01, 95% CI − 0.1 to 0.1) (Table 2). The interaction between current smoking and high EBNA-1 IgG levels remained similar in both groups when the analysis was stratified by age at EBV assessment (Table 3) and by DRB1*15:01 status (Table 4).

**Table 2:** OR with 95% CI of developing MS among subjects categorized by EBNA1 status and smoking.

EBNA-1 IgG	Smoking	ca/co^a^	OR (95% CI)^b^	OR (95% CI)^c^	AP (95% CI)
Low	Never	689/1656	1.0 (reference)	1.0 (reference)	
Low	Ever	808/1389	1.4 (1.3 to 1.6)	1.5 (1.3 to 1.7)	
High	Never	2085/1642	3.1 (2.7 to 3.4)	2.7 (2.4 to 3.0)	
High	Ever	2758/1532	4.4 (3.9 to 4.9)	3.8 (3.4 to 4.3)	0.2 (0.1 to 0.3)
Low	Never	689/1656	1.0 (reference)	1.0 (reference)	
Low	Current	505/814	1.5 (1.3 to 1.7)	1.6 (1.4 to 1.8)	
High	Never	2085/1642	3.1 (2.7 to 3.4)	2.7 (2.4 to 3.0)	
High	Current	1820/887	4.9 (4.4 to 5.5)	4.4 (3.9 to 5.0)	0.3 (0.2 to 0.4)
Low	Never	689/1656	1.0 (reference)	1.0 (reference)	
Low	Past	303/575	1.3 (1.1 to 1.5)	1.4 (1.1 to 1.6)	
High	Never	2085/1642	3.1 (2.7 to 3.4)	2.7 (2.4 to 3.0)	
High	Past	938/645	3.6 (3.1 to 4.1)	3.1 (2.7 to 3.6)	0.01 (− 0.1–0.1)

AP with 95% CI between high EBNA1 IgG and smoking.

^a^Number of exposed cases and controls

^b^Adjusted for age, sex, residential area, study, and ancestry

^c^Adjusted for age, sex, residential area, study, ancestry, infectious mononucleosis, adolescent body mass index, DRB1*1501, DRB1*0301, DRB1*1303, DRB1*0801, A*0201, B*4402, B*3801, B*5501, DQA1*0101, DQB1*0302, DQB1*0301, homozygote correction for DRB1*1501, DRB1*0301, and A*0201.

**Table 3:** OR with 95% CI of developing MS among subjects categorized by EBNA1 status and smoking, stratified by age at EBV assessment.

EBNA-1 IgG	Smoking	EBV assessment at age < 50 years			EBV assessment at age ≥ 50 years		
		ca/co^a^	OR (95% CI)^b^	OR (95% CI)^c^	ca/co^a^	OR (95% CI)^b^	OR (95% CI)^c^
Low	Never	387/1045	1.0 (reference)	1.0 (reference)	302/611	1.0 (reference)	1.0 (reference)
Low	Current	223/387	1.6 (1.3–1.9)	1.6 (1.3–2.0)	282/427	1.3 (1.1–1.6)	1.4 (1.1–1.7)
High	Never	1227/1005	3.3 (2.9–3.8)	3.0 (2.5–3.4)	858/637	2.7 (2.3–3.2)	2.4 (2.0–2.8)
High	Current	836/426	5.3 (4.5–6.3)	4.6 (3.9–5.5)	984/461	4.3 (3.6–5.1)	3.9 (3.2–4.7)
				AP 0.3 (0.1–0.4)			AP 0.3 (0.2–0.4)

AP with 95% CI between high EBNA1 IgG and smoking.

^a^Number of exposed cases and controls

^b^Adjusted for age, sex, residential area, study, and ancestry

^c^Adjusted for age, sex, residential area, study, ancestry, infectious mononucleosis, adolescent body mass index, DRB1*1501, DRB1*0301, DRB1*1303, DRB1*0801, A*0201, B*4402, B*3801, B*5501, DQA1*0101, DQB1*0302, DQB1*0301, homozygote correction for DRB1*1501, DRB1*0301, and A*0201.

**Table 4:** OR with 95% CI of developing MS among subjects categorized by EBNA1 status and smoking, stratified by DRB1*15:01 status.

EBNA-1 IgG	Smoking	DRB1*15:01 positive subjects			DRB1*15:01 negative subjects		
		ca/co^a^	OR (95% CI)^b^	OR (95% CI)^c^	ca/co^a^	OR (95% CI)^b^	OR (95% CI)^c^
Low	Never	329/401	1.0 (reference)	1.0 (reference)	360/1255	1.0 (reference)	1.0 (reference)
Low	Current	223/192	1.4 (1.1–1.8)	1.5 (1.2–1.9)	282/622	1.6 (1.3–1.9)	1.6 (1.3–2.0)
High	Never	1306/526	3.0 (2.5–3.6)	2.9 (2.4–3.5)	779/1116	2.5 (2.1–2.8)	2.5 (2.2–3.0)
High	Current	1100/272	4.9 (4.1–6.0)	4.9 (4.0–6.0)	720/615	4.0 (3.5–4.8)	4.1 (3.5–4.9)
				AP 0.3 (0.1–0.4)			AP 0.3 (0.1–0.4)

AP with 95% CI between high EBNA1 IgG and smoking.

^a^Number of exposed cases and controls

^b^Adjusted for age, sex, residential area, study, and ancestry; ^c^Adjusted for age, sex, residential area, study, ancestry, adolescent body mass index, past IM, DRB1*0301, DRB1*1303, DRB1*0801, A*0201, B*4402, B*3801, B*5501, DQA1*0101, DQB1*0302, DQB1*0301, homozygote correction for DRB1*1501, DRB1*0301, and A*0201.

A significant additive interaction also occurred between current smoking and IM history (Table 5).

**Table 5:** OR with 95% CI of developing MS among subjects categorized by IM history and smoking, stratified by EBNA1 IgG status.

IM history	Smoking	ca/co^a^	OR (95% CI)^b^	OR (95% CI)^c^	AP (95% CI)
No	Never	1989/2625	1.0 (reference)	1.0 (reference)	
No	Ever	2518/2304	1.4 (1.3 to 1.6)	1.5 (1.3 to 1.6)	
Yes	Never	380/289	1.8 (1.5 to 2.1)	1.7 (1.5 to 2.1)	
Yes	Ever	429/213	2.7 (2.3 to 3.2)	2.5 (2.0 to 3.0)	0.1 (− 0.1 to 0.3)
No	Never	1989/2625	1.0 (reference)	1.0 (reference)	
No	Current	1624/1371	1.5 (1.4 to 1.7)	1.6 (1.4 to 1.7)	
Yes	Never	380/289	1.8 (1.5 to 2.1)	1.8 (1.5 to 2.1)	
Yes	Current	269/110	3.2 (2.6 to 4.1)	3.0 (2.3 to 3.8)	0.2 (0.004 to 0.4)
No	Never	1989/2625	1.0 (reference)	1.0 (reference)	
No	Past	894/933	1.3 (1.2 to 1.5)	1.3 (1.2 to 1.5)	
Yes	Never	380/289	1.8 (1.5 to 2.1)	1.7 (1.5 to 2.1)	
Yes	Past	160/103	2.1 (1.7 to 2.8)	1.9 (1.5 to 2.6)	− 0.06 (− 0.4 to 0.3)

AP with 95% CI between past IM and smoking.

^a^Number of exposed cases and controls

^b^Adjusted for age, sex, residential area, study, and ancestry

^c^Adjusted for age, sex, residential area, study, ancestry, adolescent body mass index, EBNA1 status, DRB1*1501, DRB1*0301, DRB1*1303, DRB1*0801, A*0201, B*4402, B*3801, B*5501, DQA1*0101, DQB1*0302, DQB1*0301, homozygote correction for DRB1*1501, DRB1*0301, and A*0201. Adjusted for age, sex, residential area, study, ancestry, adolescent body mass index, DRB1*1501, DRB1*0301, DRB1*1303, DRB1*0801, A*0201, B*4402, B*3801, B*5501, DQA1*0101, DQB1*0302, DQB1*0301, homozygote correction for DRB1*1501, DRB1*0301, and A*0201.

All main findings remained significant when the studies were analyzed separately (data not shown). Our results remained almost identical after carrying out the analyses on the multiple imputed data^25^ (data not shown). Our results also remained stable when smoking was considered 2 years prior to the index year (data not shown).

## Discussion

Our results demonstrate a significant interaction on the additive scale between current smoking and both high EBNA-1 IgG levels and IM history, with regard to MS risk. The interactions remained similar regardless of DRB1*15:01 status.

There may be several explanations to why results from previous studies on the interplay between smoking and aspects of EBV infection have been conflicting. The studies have often been small with limited power to detect interactions of moderate size. In all studies but one, smoking was classified as ever or never smokers^4–7,9^. If interactions only occur between current smoking and EBV infection, the likelihood of a negative finding increases if the past smokers are classified as ever smokers together with the current smokers, especially if the proportion of past smokers is large. The last study, performed in Sweden, used cotinine levels as a measure of smoking^8^. However, cotinine levels are also high in users of smokeless tobacco and nicotine, which when considered in isolation of the products of combustion, has been associated with reduced MS risk. In Sweden, where the use of smokeless tobacco is a common habit, cotinine levels may not be a good proxy for smoking. Some studies, including the largest one^6^, only assessed multiplicative interaction whereas additive interaction was not considered. Since the logistic regression model is inherently multiplicative, presence of an interaction term implies departure from multiplicativity, which has no direct relevance for the issue of whether or not sufficient-cause interaction is present^17^. Taken together, this may explain previous contradictory results.

After primary EBV infection, the virus remains in a latent phase in resting memory B cells. The virus may be reactivated during periods of environmental stress, whereby the EBV antibodies against viral antigens become elevated. Smokers have higher levels of anti-EBV antibodies^26,27^ and higher EBV viral load^28,29^ compared to non-smokers, and several studies indicate that smoking may trigger EBV reactivation^30,31^.

Smoking alters the development and function of both innate and adaptive immune cells, and leads to pro-inflammatory responses and dysfunction of immune cells^32^. Memory B-cells play an important role in MS pathogenesis since they are reservoirs for EBV latency. They are antigen-presenting cells, which may activate auto-aggressive T-cells against CNS antigens. Several MS-associated risk alleles responsible for the regulation of B-cell functions have been identified^33^. Smoking increases the frequency of memory B cells and lowers regulatory B cell numbers^32,34^. Smoking also has anti-estrogen effect which may alter survival and activation of autoreactive B cells and skew the immune system toward autoimmunity^35^.

The components in cigarette smoke also affect the immune system barrier function and may promote the migration of autoreactive immune cells into the CNS^36^. Furthermore, epigenetic alterations are induced by smoking, including extensive genome-wide changes in DNA methylation^37,38^. Smoking-associated DNA methylation and changes in gene expression among immune cell types have been identified and may contribute to EBV reactivation^39^.

Both HLA and non-HLA genes are involved in controlling EBV infection^40,41^, and both high EBNA-1 IgG levels and past IM interact with carriage of DRB1*15:01 and absence of HLA-A*02:01^21^. The DRB1*15:01 allele affects the humoral response to EBV and genetic differences in the class I locus have been shown to influence both the outcome of primary EBV infection and the viral persistence^42,43^. Smoking has been shown to interact with the same MS-associated HLA genes^20^. Altogether, these findings point towards a complex interaction between smoking and EBV, which is affected by genetic constitution and probably also by other environmental factors.

Individuals with MS often show enhanced response to several epitopes in EBNA-1. In the present study, we defined high EBNA-1 IgG levels as increased reactivity against peptides that spanned aa 385–420 since this segment has been identified as the primary EBNA-1 fragment for which antibody response is associated with MS risk^15^. Whether enhanced reactivity to other unique EBNA-1 epitopes interact with smoking to increase MS risk is unclear.

The term interaction is somewhat confusing since interaction depends on the scale of measurement, i.e. presence of interaction on one scale does not necessarily presence of interaction on the other. E.g. presence of no interaction between two risk factors (with OR larger than 1) on the additive scale imply negative interaction on the multiplicative scale and presence of no interaction between two risk factors on the multiplicative scale implies that there is interaction on the additive scale. It has been shown that interaction on the additive scale is more informative from a public health perspective. Further, the sufficient-cause concept that was developed by Rothman and later expanded by Vanderwheele has improved the understanding between disease causation and interaction, in that presence of interaction between two causal factors on the additive scale imply that there exists a pathway towards disease where the presence of both risk factors are needed.

Both EIMS and GEMS were designed as case–control studies and information on exposures and lifestyle factors was collected retrospectively. There could be a potential recall bias in this design and we made great efforts to obtain exposure information from cases and controls in an identical way. The questionnaire comprised a large number of questions regarding many environmental and lifestyle habits, and no section in the questionnaire was given main focus.

In both studies, selection bias was minimized by the population-based design. The health care system in Sweden provides equal free of charge access to medical services for all citizens, and MS cases are referred to neurological units, making them eligible to be part of the studies. Although the relatively high proportion of non-responders among the controls may introduce selection bias, it is probably modest since the prevalence of life style factors, such as smoking and alcohol consumption, among the controls was consistent with that of the general population in similar ages^44^.

Blood samples for genetic and serologic analyses were not available for a substantial proportion of cases and controls, which were excluded from the study. Among both cases and controls, smokers were more prone to provide blood. However, the OR of MS associated with smoking was the same among those who did and did not donate blood. There is no reason to believe that DRB1*15:01 status or EBNA-1 IgG levels would differ between those who donated blood and those who did not. We thus consider it unlikely that our findings would be affected by bias to a large extent.

In conclusion, current smoking increases EBNA-1 IgG levels and acts synergistically with both aspects of EBV infection to increase MS risk.

## Supplementary information

Supplementary file1 (PDF 196 kb)

## Data Availability

Anonymized data will be shared by request from any qualified investigator that wants to analyze questions that are related to the published article.
