# Supplementary material for: Smoking and Epstein–Barr virus infection in multiple sclerosis development
Source: Sci Rep. 2020 Jul 3;10:10960. doi: 10.1038/s41598-020-67883-w (PMC7335184; doi:10.1038/s41598-020-67883-w)
Supplement: Supplementary file 1 — Supplementary file1 (PDF 196 kb) [file 41598_2020_67883_MOESM1_ESM.pdf]

Smoking and Epstein-Barr virus infection in multiple sclerosis development

Anna Karin Hedström[1-2], Jesse Huang[2-3], Nicole Brenner[4], Julia Butt[4], Jan Hillert[2-3], Tim Waterboer[4], Ingrid Kockum[2-3], Tomas Olsson[2-3]", Lars Alfredsson[1-2]"

Online Resource 1. Characteristics of cases and controls, overall and by smoking status.

	Cases				Controls			
	Total	Current smokers	Past smokers	Never smokers	Total	Current smokers	Past smokers	Never smokers
Women, n (%)	4596 (72)	1649 (71)	889 (72)	2058 (74)	4739 (76)	1334 (78)	938 (77)	2467 (75)
Men, n (%)	1744 (28)	676 (29)	352 (28)	716 (26)	1480 (24)	367 (22)	282 (23)	831 (25)
Mean BMI, kg/m^2 (SD)	22.0 (4.6)	22.0 (5.5)	22.1 (5.0)	22.1 (3.5)	21.8 (4.4)	21.7 (4.4)	21.5 (2.8)	21.9 (4.8)
Mean anti-EBNA1 IgG level (SD)	7669 (3385)	7918 (3424)	7657 (3417)	7466 (3325)	5510 (3488)	5697 (3599)	5628 (3399)	5369 (3457)
Median anti-EBNA1 IgG level	7775	8021	7754	7561	5620	5717	5794	5500
Past IM, n (%)	809 (13)	269 (12)	160 (13)	380 (14)	502 (8.1)	110 (6.5)	103 (8.4)	289 (8.8)
DRB1*15:01, n (%)	3654 (58)	1323 (57)	696 (56)	1635 (59)	1752 (28)	464 (28)	361 (30)	927 (28)
Total	6340	2325	1241	2774	6219	1701	1220	3298

Online Resource 2. Characteristics of cases and controls who were included and excluded in the study, by EIMS and GEMS.

	EIMS				GEMS			
	Included		Excluded		Included		Excluded	
	Cases	Controls	Cases	Controls	Cases	Controls	Cases	Controls
Women, n (%)	1467 (73)	1839 (75)	601 (70)	2530 (69)	3129 (72)	2900 (77)	1284 (73)	1088 (69)
Men, n (%)	554 (27)	610 (25)	258 (30)	1143 (31)	1190 (28)	870 (23)	482 (27)	499 (31)
Ever smoking, n (%)	1074 (53)	1093 (45)	442 (51)	1496 (41)	2489 (58)	1827 (48)	917 (52)	706 (44)
Mean BMI, kg/m^2 (SD)	22.6 (3.9)	21.8 (3.1)	22.5 (3.7)	22.0 (3.6)	21.8 (4.9)	21.8 (5.0)	22.0 (3.8)	21.7 (3.0)
Mean anti-EBNA1 IgG level (SD)	7495 (3025)	5254 (3278)	-	-	7750 (3528)	5676 (3608)	-	-
Median anti-EBNA1 IgG level	7758	5457	-	-	7789	5715	-	-
Past IM, n (%)	345 (17)	252 (10)	157 (18)	370 (10)	464 (11)	250 (6.6)	252 (14)	115 (7.3)
DRB1*15:01, n (%)	1111 (55)	681 (28)	-	-	2543 (59)	1071 (28)	-	-
Men age at disease onset (SD)	34.4 (10.6)	-	35.2 (10.6)	-	34.0 (22.8)	-	31.7 (10.3)	-
Men age at study inclusion (SD)	38.7 (11.3)	-	39.0 (11.2)	-	53.6 (11.8)	-	46.1 (12.9)	-
Total	2021	2449	859	3673	4319	3770	1766	1587

Additionally, 71 cases and 51 controls, for which exposure information was not available, were excluded from GEMS.
